# Identification and transfer of a new *Pm21* haplotype with high genetic diversity and a special molecular resistance mechanism

**DOI:** 10.1007/s00122-023-04251-y

**Published:** 2023-01-19

**Authors:** Zhenpu Huang, Jiaqian Liu, Xiangqian Lu, Yifei Guo, Yueying Li, Yangqi Liu, Ruiqi Zhang, Liping Xing, Aizhong Cao

**Affiliations:** 1 National Key Laboratory of Crop Genetics and Germplasm Enhancement, Cytogenetics Institute, Nanjing Agricultural University/JCIC-MCP, Nanjing, 210095 China; 2 State Key Laboratory for Quality and Safety of Agro-Products, Institute of Plant Virology, Ningbo University, Ningbo, 315000 China

## Abstract

**Key message:**

A new functional* Pm21* haplotype,* Pm21(8#)*, was cloned from the new wheat-*H. villosa* translocation line T6VS(8#)·6DL, which confers the same strong resistance to powdery mildew through a different resistance mechanism.

**Abstract:**

Broad-spectrum disease resistance genes are desirable in crop breeding for conferring stable, durable resistance in field production. *Pm21(4#)* is a gene introduced from wild *Haynaldia villosa* into wheat that confers broad-spectrum resistance to wheat powdery mildew and has been widely used in wheat production for approximately 30 years. The discovery and transfer of new functional haplotypes of *Pm21* into wheat will expand its genetic diversity in production and avoid the breakdown of resistance conferred by a single gene on a large scale. *Pm21(4#)* previously found from T6VS(4#)·6AL has been cloned. In this study, a new wheat-*H*. *villosa* translocation, T6VS(8#)·6DL, was identified. A new functional *Pm21* haplotype, designated *Pm21(8#)*, was cloned and characterized. The genomic structures and the splicing patterns of *Pm21(4#)* and *Pm21(8#)* were different, and widespread sequence diversity was observed in the gene coding region and the promoter region. In the field, *Pm21(8#)* conferred resistance to *Blumeria graminis* f. sp. *tritici* (*Bgt*), similar to *Pm21(4#)*, indicating that *Pm21(8#)* was also a resistance gene. However, *Bgt* development during the infection stage was obviously different between *Pm21(4#)-* and *Pm21(8#)-*containing materials under the microscopic observation. *Pm21(4#)* inhibited the formation of haustoria and the development of hyphae in the initial infection stage, while *Pm21(8#)* limited the growth of hyphae and inhibited the formation of conidiophores in the late infection stage. Therefore, *Pm21(8#)* is a new functional *Pm21* haplotype that provides a new gene resource for wheat breeding.

**Supplementary Information:**

The online version contains supplementary material available at 10.1007/s00122-023-04251-y.

## Introduction

Wheat powdery mildew is a worldwide fungal disease caused by the obligate biotrophic plant pathogen *Blumeria graminis* f. sp. *tritici* (*Bgt*), which belongs to the Ascomycotas group Erysiphales in the fungal kingdom (Takamatsu 2004). The disease can occur in leaves, stems and spikes, leading to a severe effect on wheat growth and a great decease in grain yield (Griffey et al. 1993; Randhawa et al. 2019). Therefore, the breeding and cultivation of powdery mildew resistant varieties is a highly efficient and eco-friendly way to ensure food security in major wheat-producing countries (Randhawa et al. 2019; Dreiseitl 2021). The identification of powdery mildew (*Pm*) genes and the development of germplasms containing *Pm* genes are the basis for breeding resistant varieties. To date, more than 100 *Pm* genes have been explored, among which 25 came from the wild relatives of wheat (Zhu et al. 2022), but only a few of these genes have been used in disease resistance breeding. Most of the *Pm* genes confer race-specific resistance, which is short-lived due to the rapid changes in virulence of the *Bgt* population (Zeng et al. 2014; Jin et al. 2021; Wu et al. 2021). In addition, *Pm* genes from the wild species are usually accompanied by unfavorable traits; therefore, a few *Pm* genes from the wild species, such as *Pm8* from *Secale cereale* and *Pm21* from *Haynadia villosa*, have been widely used in wheat production (Wu et al. 2021). Therefore, the identification and utilization of *Pm* genes with broad-spectrum, strong effect are the most urgent and challenging tasks.

In China, wheat powdery mildew is one of the most devastating fungal diseases in all of the ecological areas of wheat production. Resistance genes have played an important role in preventing powdery mildew in wheat. *Pm21* confers high-level, broad-spectrum resistance to powdery mildew, which was transferred from *H. villosa* to wheat through the cytogenetical development of the wheat-*H. villosa* translocation T6VS·6AL (Chen et al. 1995). The T6VS·6AL line has been widely used and has made significant contributions to the disease control in the main wheat-producing regions of China. In the southwestern wheat planting area, the proportions of elite lines carrying *Pm21* are high, and in the middle and lower reaches of the Yangtze River, approximately 28.3% of commercial varieties carry the *Pm21* gene (Wu et al. 2021). According to the statistical data from 2003 to 2012, the planting area of *Pm21*-carrying varieties was more than 4 million hectares (Xing et al. 2018). In recent years, due to the increase in the number of released varieties carrying *Pm21*, the planting area has also significantly expanded. There have been many cases in which resistance genes, including *Pm1*, *Pm3a*, *Pm3b*, *Pm3c*, *Pm3f*, *Pm5*, *Pm7* and *Pm8*, have been overcome after large-scale application due to the emergence of new virulent isolates (Wang et al. 2005). For example, *Pm8*, a resistance gene transferred to wheat through the development of the wheat-*S. cereale* translocation T1RS·1BL, has played a major role in the prevention of powdery mildew worldwide (Hsam et al. 1997). In China, the percentage of *Pm8*-carrying varieties was as high as 70% in the 1980s (Zhou et al. 2004). However, *Pm8* lost its resistance in most areas due to large-scale application in the end of 1980s, leading to the extreme lack of powdery mildew resistant varieties all over the country (Sheng et al. 1995). Currently, the large-scale, frequent use of a single *Pm21* gene in some ecological areas of China is imposing strong selection pressure on *Bgt*, which will probably lead to the resistance breakdown. Therefore, it is particularly important to explore and reasonable layout new genes or new functional haplotypes of known genes with broad-spectrum resistance to powdery mildew.

*Haynaldia villosa* (2*n* = 14, VV), an annual cross-pollinated diploid species indigenous to the Mediterranean and Caucasus regions, provides a valuable tertiary gene pool for wheat improvement. More than 300 *H. villosa* accessions have been collected from their native habitats, and these accessions show high genetic diversity and displaying extremely high value for the improvement of tolerance to biotic and abiotic stresses and agronomic traits (De Pace et al. 2011; Chen et al. 2002; Grądzielewska et al. 2006). However, only a few accessions have been used to develop wheat-*H. villosa* alien chromosome lines, and even fewer *H. villosa* accessions have played roles in successful wheat breeding through the developed germplasms. According to De Pace (2011), seven accessions have been successively used to synthesize hexaploid amphidiploids, including Hv(1#) (Sears 1953), Hv(2#) (Lukaszewski 1988), Hv(3#) (Urbano 1988), Hv(4#) (Liu et al. 1988; Chen et al. 1995), Hv(5#) (Chen et al. 1996; Li et al. 2005), Hv(6#) (Ma et al. 1997) and Hv(7#) (De Pace 2001).

Previously, the total number of *Pm* genes was reported to be more than 100 because of multiple haplotypes at some loci, including *Pm1*, *Pm2*, *Pm3*, *Pm4*, *Pm5* and *Pm24*, etc. Genetic diversity provides an opportunity to explore new functional resistance haplotypes for breeding. For example, more than 17 *Pm3* haplotypes have been identified, and some haplotypes still show high breeding value, even though some haplotypes have completely lost their ability to confer resistance (Brunner et al. 2012; Koller et al. 2018). Therefore, the mining of new functional haplotypes from previously identified loci is a promising method for addressing resistance gene deficiency.

Xing et al. (2018) cloned *Pm21(4#)* from Hv(4#) via combinational approaches, and Li et al. (2020) cloned *Pm21(5#)* from Hv(5#) through a homology-based approach. *H. villosa* displays high genetic diversity, and more importantly, the *Pm21* haplotypes show high sequence polymorphism. Therefore, it is possible to identify new *Pm21* haplotypes conferring resistance mediated by alternative molecular mechanisms.

In this study, to identify a novel functional *Pm21* haplotype, a new wheat-*H. villosa* translocation line, T6VS(8#)·6DL, was developed and characterized. The high and broad-spectrum resistance of T6VS(8#)·6DL is mediated by *Pm21(8#)*. However, *Pm21(8#)* shows dramatic sequence polymorphism, different transcriptional patterns, and a special resistance mechanism. Therefore, the identified *Pm21(8#)* haplotype, with high genetic diversity and a distinct resistance mechanism, provides a new resistance haplotype and new resistant germplasm for breeding.

## Materials and methods

### Plant materials

The wheat variety Nannong9918, named as T6VS(4#)·6AL, was developed by the Cytogenetic Institute of Nanjing Agricultural University (CINAU). The wheat variety Yangmai 22, named as T6VS(5#)·6DL, was introduced from the Lixiahe Agricultural Research Institute of Jiangsu Academy of Agricultural Sciences. T6VS (8#)·6DL was developed by CINAU through chromosome engineering. The 6VS fragments of T6VS(4#)·6AL, T6VS(5#)·6DL and T6VS(8#)·6DL were derived from different *H. villosa* accessions, and *Pm21* haplotypes display high diversity in their nucleotide and protein sequences. Powdery mildew-susceptible *cv.* Nannong0686 was developed by CINAU and used as the recurrent parent to develop the Nannong0686-*Pm21(8#)* and Nannong0686-*Pm21(5#)* lines.

### Inoculation of *Bgt* and evaluation of powdery mildew resistance

The powdery mildew resistance of T6VS(8#)·6DL was evaluated at the adult stage in the field and at the seedling stage in the greenhouse. In the field, T6VS(8#)·6DL was infected by the local mixed races of *Bgt* at the Baima Experimental Station of Nanjing Agricultural University (Nanjing, China). The *Bgt* isolate E26 was obtained from Dr. Yilin Zhou of the Chinese Academy of Agricultural Sciences and maintained on seedlings of the susceptible variety Nannong0686 in the greenhouse under a 14/10-h light/dark photoperiod (24/18 °C temperature, 70% relative humidity). In the greenhouse, *Bgt* inoculation was performed by spraying fresh conidiospores of E26 from the susceptible host Nannong0686 onto the seedling leaves. T6VS(4#)·6AL, T6VS(5#)·6DL and T6VS (8#)·6DL plants at the two-leaf stage were inoculated with E26 and harvested at different time points to observe *Bgt* development, detect H_2_O_2_ accumulation and analyze gene expression. The infection types (ITs) were scored on a 0–4 scale, with 0 indicating no visible symptoms; 1 indicating minute colonies with few conidia; 2 indicating colonies with moderately developed hyphae, but few conidia; 3 indicating colonies with well-developed hyphae and abundant conidia, but colonies not joined together; and 4 indicating colonies with well-developed hyphae and abundant colonies that are mostly joined together (Liu et al. 1999).

### Silencing of *Pm21(8#) *by barley stripe mosaic virus induced gene silencing (BSMV-VIGS)

BSMV-VIGS was performed as described by Hu et al. (2018) and Xing et al. (2018). *Pm21(8#)* was silenced by BSMV-VIGS in T6VS(8#)·6DL to evaluate the powdery mildew resistance function. Previously, the 225 bp fragment specific to *Pm21(4#)* was amplified and inserted into the γ-strain of BSMV to produce the recombinant vector for silencing *Pm21(4#)* (Xing et al. 2018), and this recombinant vector was also used for silencing *Pm21(8#)* in this study. The second fully expanded leaves were infected with the in vitro-transcribed virus BSMV:*Pm21(8#)*, and BSMV:*TaPDS-* and BSMV:γ-infected leaves were used as controls. Then, the fourth fully expanded leaves with clear virus infection symptoms were used to evaluate powdery mildew resistance and to analyze the target gene silencing efficiency. The leaves were placed on the 20 mg/l 6-BA agar medium and inoculated with fresh *Bgt* E26 in a greenhouse with a 14-h light (25 °C)/10-h dark (18 °C). Then the resistance level was recorded at 7 days after *Bgt* inoculation. Leaves were bleached with ethanol:acetic acid (3:1) and stained with Coomassie blue (6 mg/ml) to observe fungal development under an Olympus BX-60 microscope (Olympus, Tokyo, Japan). The gene silencing efficiency was analyzed by qRT‒PCR on an LC 480II instrument (Roche, Colorado Springs, CO, USA).

### Cloning and sequence analysis of *Pm21* haplotypes

The genomic sequences and transcripts of *Pm21(8#)* were obtained according to the sequences of *Pm21(4#)* through homology-based cloning. The nucleotide and codon protein sequences of *Pm21(4#)*, *Pm21(5#)* and *Pm21(8#)*, were aligned by using DNAMAN software (https://www.lynnon.com/). The *cis*-element prediction in promoters from the three genes was performed on PlantCare (https://www.plantcaretools.com/). The putative domains of the cloned genes were analyzed using SMART (http://smart.embl-heidelberg.de/) and visualized with TBtools software (https://github.com/CJ-Chen/TBtools). The phylogenetic tree was constructed by using MEGA7 software base on neighbor-joining algorithms, and bootstrapping was performed 1000 times to obtain support values for each branch (Kumar et al. 2016). The tree was visualized using iTOL (Letunic et al. 2021). The conserved motifs of the NLR protein were annotated using MAST (version 4.9.1) (Bailey et al. 2009) and the ‘motif 1’ to ‘motif 20’ were defined by Jupe (2012).

### Genomic in situ hybridization (GISH) and florescence in situ hybridization (FISH)

To identify the introgressed 6VS chromosomal fragment in the translocation lines, genomic in situ hybridization (GISH) and fluorescence in situ hybridization (FISH) were performed following Du et al. (2017), using root-tip cells at mitotic metaphase with AMP soaking and nitrous oxide treatment as described by Komuro et al. (2013). Total genomic DNA of *H. villosa* (91C43) labeled with 5’ FAM was used as the GISH probe, which produced green fluorescence signals. Oligo-pAs1-1 and oligo-pAs1-4 labeled with 5’ TAMRA in a mixture were used as the probes for FISH analysis, which produced red fluorescence signals. Moreover, the wheat chromosomes were counterstained with DAPI and visualized based on blue fluorescence. Oligo-painting as described by Wang et al. (2017), using pAs1-1, pAs1-3, pAs1-4, pAs1-6, AFA-3, AFA-4, pSc119.2–1 and (GAA)10 as the mixed probes, was performed to identify the polymorphism of these repeat sequences among the three 6VS chromosome arms. The hybridized slides were observed under an Olympus BX60 fluorescence microscope with a CCD camera DP72 (Olympus, Tokyo, Japan) for image acquisition, and individual chromosomes with hybridization signals were cropped using the Adobe Photoshop software CS 7.0 (Adobe Systems, San Jose, CA, USA).

### Development of molecular markers and analysis of the genetic population

Molecular markers specific to *Pm21(8#)*, including *Pm21_588*, *Pm21_1178* and *Pm21_2683*, were developed according to the sequences comparison of *Pm21(8#)* with *Pm21(5#)* and *Pm21(4#)*, and the primers are listed in Table S1. For the allelic testing of *Pm21(5#)* and *Pm21(8#)*, an F_2_ population was derived by crossing T6VS(5#)·6DL and T6VS(8#)·6DL. The *Pm21* haplotypes of the two translocation parents, which were recombinant plants homozygous for *Pm21(5#)* and *Pm21(8#)* (designated F_2_-Pm21(5#) and F_2_-Pm21(8#), respectively) were analyzed based on specific markers. PCR amplification was performed using a T100TM Thermal Cycler (Bio-Rad Laboratories, Hercules, California, USA) in a 10 μL reaction solution containing 80–100 ng of genomic DNA, 2.5 mmol L^−1^ of each dNTP, 2 μmol L^−1^ of each primer, 2.5 mmol L^−1^ MgCl_2_ and 0.2 U *Taq* DNA polymerase. The PCR cycling conditions were as follows: 94 °C for 4 min, followed by 32 cycles of 94 °C for 15 s, 58 to 60 °C for 45 s, and 72 °C for 60 s, with a final extension at 72 °C for 10 min. The PCR products were separated on 8% nondenaturing PAGE gels (Acr:Bis = 39:1) and visualized by silver staining.

### Observation of *Bgt* development and detection of H_2_O_2_ accumulation

To compare *Bgt* development and H_2_O_2_ accumulation mediated by different *Pm21* haplotypes, the third leaves of T6VS(4#)·6AL, T6VS(5#)·6DL and T6VS(8#)·6DL and plants of the selected individuals from the F_2_ population with homozygous *Pm21(5#)* and *Pm21(8#)* were inoculated with *Bgt* isolate E26 at 24, 48, 72 and 120 h after inoculation. Then, the detached leaves were bleached in ethanol:acetic acid (3:1, v/v) at the 37 °C incubator overnight and finally stained with Coomassie Brilliant Blue R250 for 5 min and examined under an Olympus BX40 microscope (Olympus, Tokyo, Japan). The leaves were also stained with DAB solution (1 mg/ml, pH 3.8) for 8 h, decolorized in ethanol:acetic acid (3:1), and observed under the same microscope. The numbers of whole cells in which H_2_O_2_ was distributed were counted in approximately 200 to 300 *Bgt*-interacted epidermal cells of each leaf in both the resistant plants and the susceptible plants, and three leaves of each plant were sampled as an experimental repetition. Three independent replicate experiments were performed, and their results were statistically analyzed using SPSS 16.0 software.

### Analysis of the haustorium index via a single-cell GUS expression assay

The single-cell transient overexpression assay was performed according to Hu et al. (2018) described. The vector *pWMB002* carrying the β-glucuronidase (*GUS*) gene driven by the ubiquitin promoter was used as the reporter of the transformed epidermal cells. Seedlings of the Nannong0686 and Nannong9918, Nannong0686-*Pm21(8#) *and Nannong0686-*Pm21(5#)* lines and the F_2_-Pm21(5#) and F_2_-Pm21(8#) lines were grown under the growth conditions with a 16-h light (18 °C)/8-h dark (16 °C) cycle and constant relative humidity of 70%. In the two-leaf stage, primary leaves were cut and placed on 20 mg/l 6-BA agar plates. The plasmid *pWMB002* was extracted with a High Purity Plasmid Extraction Kit (TIANGEN, Beijing, China), coated with the particles and delivered into wheat epidermal cells through bombardment (PDS-1000/He Bio-Rad, California, USA). The bombarded leaves were incubated in the dark at 18 °C for 4 to 6 h before high-density inoculation with *Bgt* conidiospores. The leaves were stained for GUS at 24 h after *Bgt* inoculation, and after 18–24 h of GUS staining, both the *GUS*-expressing cells and the haustoria of the *Bgt*-infected cells were visible based on blue coloration under an Olympus BX40 microscope (Olympus, Tokyo, Japan). The haustorium index was calculated as described by Hu et al. (2018). Three independent replicated experiments were carried out, and for each replicate included the examination of 100–300 successfully *GUS*-expressing cells interaction with *Bgt*. The data were statistically analyzed using SPSS 16.0 software.

## Results

### Development of the new wheat-*H. villosa* translocation T6VS(8#)·6DL

**Fig. 1:**
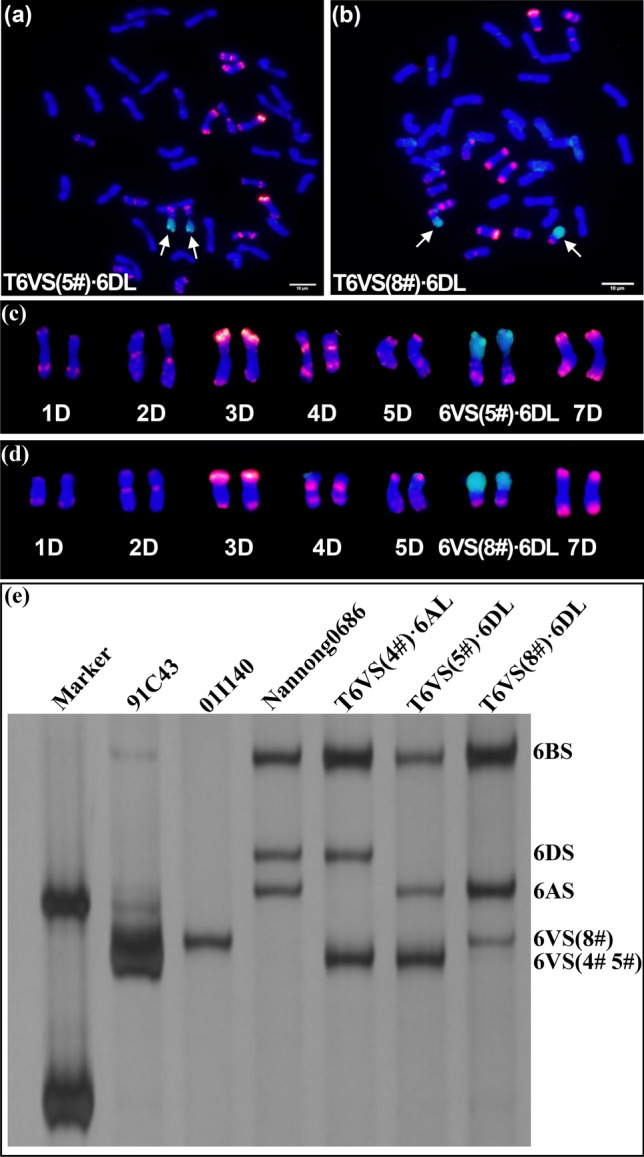
Cytogenetic identification and molecular marker analysis of the new wheat-*H. villosa* translocation T6VS(8#)·6DL, **a–b** Genomic in situ hybridization (GISH) and fluorescence in situ hybridization (FISH) analysis of T6VS(5#)·6DL and T6VS(8#)·6DL on root tip metaphase chromosomes. Green fluorescence represents GISH analysis signal obtained using the total genomic DNA of *H. villosa* as a probe, and the red fluorescence represents the FISH analysis signal obtained using oligo-pAs as a probe. Chromosomes were counterstained with DAPI (blue). The translocation chromosomes are indicated with white arrows. **c–d** GISH**-**FISH patterns of the D-genome chromosomes of the two translocation lines. The chromosomes were extracted from a and b respectively, and the two translocation chromosomes, 6VS(5#)·6DL and 6VS(8#)·6DL, were identified and are indicated. **e** PCR analysis of the codominant molecular marker CINAU15. The chromosome specific bands are labeled on the right, and the band of 6VS(8#) was different from that of 6VS(4#) or 6VS(5#) (color figure online)

Phenotypic evaluation of different accessions of *H. villosa* (2*n* = 14, VV) revealed the high morphological polymorphism, indicating high genetic diversity. *H. villosa 01I140*, Hv(8#), which originated from Italy, was selected as the male parent, and the tetraploid wheat *cv.* Zhongyin1286 was used as the female parent to synthesize *T. durum*-*H*. *villosa* amphidiploid (2*n* = 14, AABBVV) (Zhang et al. 2018). Then, the amphidiploid was used as the V genome for backcrossing with Nannong0686 to induce wheat-*H*. *villosa* translocation involved different chromosome arms of V genome. In the backcross-derived offspring, the wheat-*H*. *villosa* translocation line T6VS·6DL was identified, which showed high resistance to powdery mildew. The results of cytogenetic analysis showed that seven pairs of chromosomes displayed a red signal, indicating that they were D genome since pAs1-1 and pAs1-4 were the D-genome specific probes. However, one pair of chromosomes showed a strong green signal on the short arm indicating that they were translocated chromosome between wheat and *H. villosa* (Fig. 1a–d). Because the long arm of the translocated chromosome was 6DL, so we proposed that the short arm was 6VS, produced by the recombination between 6D and 6V. Previously, we developed a molecular marker, CINAU15, to distinguish the 6AS, 6BS, 6DS and 6VS (Cao et al. 2006). The marker analysis showed that the bands specific to 6AS, 6BS, and 6DS were amplified from Nannong0686, bands specific to 6DS, 6BS, and 6VS were amplified from the wheat-*H.villosa* translocation line T6VS(4#)·6AL, and bands specific to 6AS, 6BS, and 6VS were amplified from the wheat-*H.villosa* translocation line T6VS(5#)·6DL and T6VS(8#)·6DL (Fig. 1e). These results indicated that in T6VS(8#)·6DL, the 6DS was substituted by 6VS(8#). Interestingly, the size of the bands produced from 6VS of T6VS(8#)·6DL was different from those of T6VS (5#)·6DL and T6VS (4#)·6AL, indicating the existence of genetic diversity between different V genomes. Therefore, the newly developed germplasm T6VS(8#)·6DL is a novel wheat-*H. villosa* translocation line.

### Cytogenetic comparison of the T6VS(8#)·6DL with other translocations

Previously, two wheat-*H*. *villosa* translocation lines involving 6VS, T6VS(4#)·6AL developed by CINAU and T6VS(5#)·6DL developed by CAAS, have been reported to display complete immunity to powdery mildew. Both germplasms have been used in breeding pipelines to develop many commercial cultivars. To study whether the 6VS(4#), 6VS(5#) and 6VS(8#) display diversity at the level of cytogenetic analysis, oligo-painting was performed to identify the polymorphism of the repeat sequences among the three 6VS chromosome arms (Fig. S1a–c). The results indicated that the green signals, produced by oligo-pSc119.2–1 and oligo-(GAA)10, displayed similar distribution patterns in 6VS(4#), 6VS(5#) and 6VS(8#). However, the red signals, produced by oligo-pAs1-1, oligo-pAs1-3, oligo-pAs1-4, oligo-pAs1-6, oligo-AFA-3 and oligo-AFA-4, showed different distribution patterns in 6VS(4#) from those in 6VS(5#) and 6VS(8#) (Fig. S1d–e). Therefore, the results from both molecular marker and cytogenetic marker analyses identified diversity among different V genomes.

### Identification of a new *Pm21* haplotype in T6VS(8#)·6DL

#### Evaluation of the powdery mildew resistance of T6VS(8#)·6DL

**Fig. 2:**
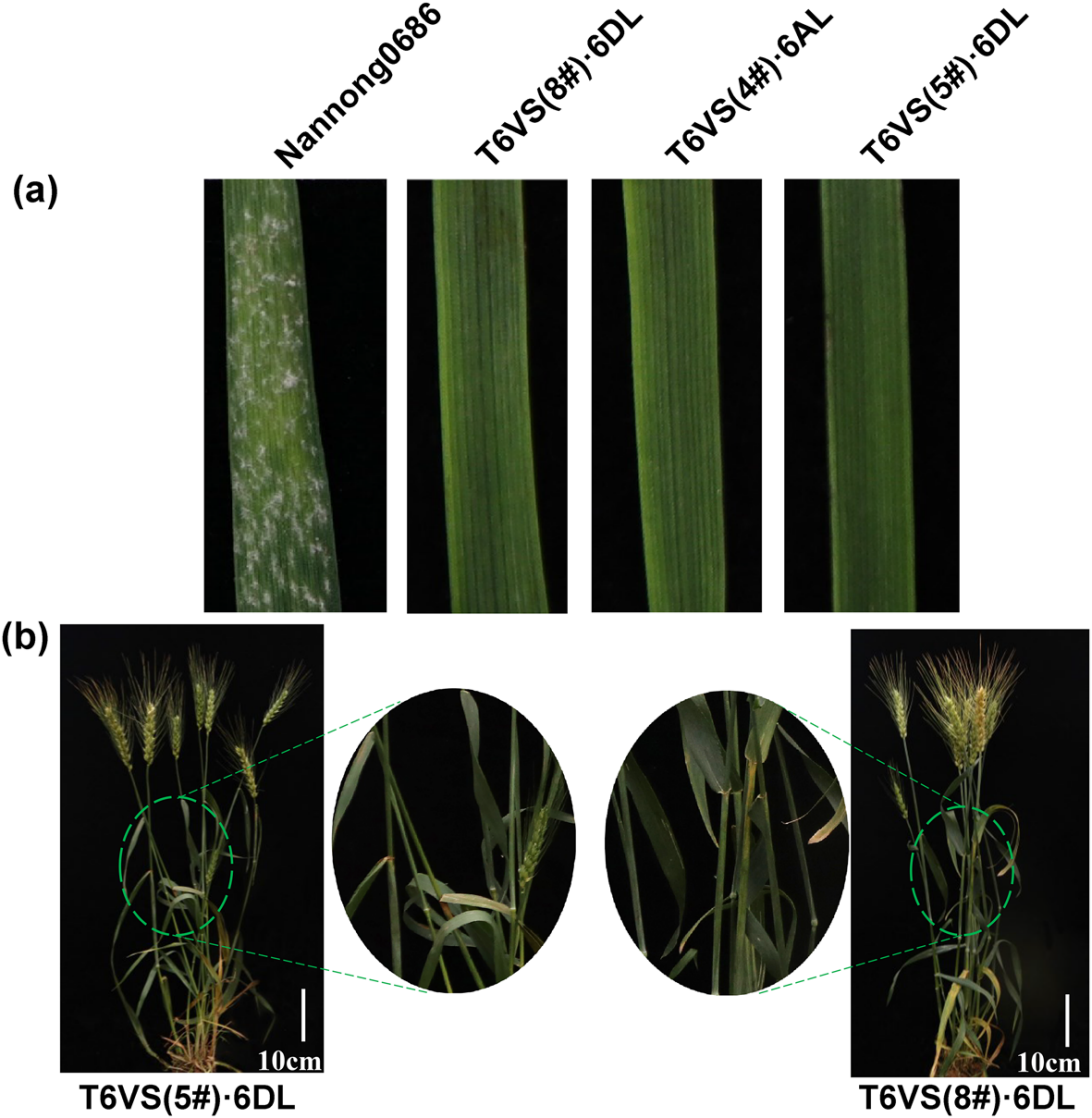
Evaluation of the powdery mildew resistance in the wheat-*H. villosa* translocation line T6VS(8#)·6DL, **a** Powdery mildew resistance evaluation in T6VS(8#)·6DL at the seedling stage in the greenhouse, using T6VS(4#)·6AL and T6VS(5#)·6DL as the resistant controls and Nannong0686 as the susceptible control. **b** Powdery mildew resistance evaluation in T6VS(8#)·6DL at the adult plant stage in the field, using T6VS(5#)·6DL as the resistant control

The resistance of T6VS(8#)·6DL to powdery mildew was evaluated using the naturally mixed *Bgt* isolates in the field (Fig. 2b), and T6VS(8#)·6DL showed complete immunity to powdery mildew both in the seedling stage and the adult plant stage. Then, the resistance level of T6VS(8#)·6DL to powdery mildew was compared to that of T6VS (4#)·6AL and T6VS(5#)·6DL in the greenhouse (Fig. 2a). The three translocation lines showed a high level of resistance to the *Bgt* isolate E26, without visible colonies on their leaf surfaces (Fig. 2). Therefore, T6VS(8#)·6DL showed the same high level of resistance to powdery mildew as T6VS(4#)·6AL and T6VS(5#)·6DL.

#### Identification of a new *Pm21* allele, *Pm21*(*8#*), in T6VS(8#)·6DL

Since both T6VS(4#)·6AL and T6VS(5#)·6DL contain functional *Pm21* alleles, designated *Pm21(4#)* and *Pm21(5#)*, respectively, it was hypothesized that there was a functional *Pm21(8#)* allele was present in T6VS(8#)·6DL. T6VS(8#)·6DL was crossed with T6VS(5#)·6DL to produce an F_2_ population for the allelism test of *Pm21(5#)* in T6VS(5#)·6DL with *Pm21(8#)* in the T6VS (8#)·6DL. All the F_2_ individuals showed a high level of resistance similar to those of both parents, with no visible colonies, and no susceptible individual was produced due to chromosome recombination (Fig. S2). Therefore, this indicates that a new *Pm21* allele, *Pm21(8#)*, is present in T6VS(8#)·6DL, as inferred from the genetic analysis.

**Fig. 3:**
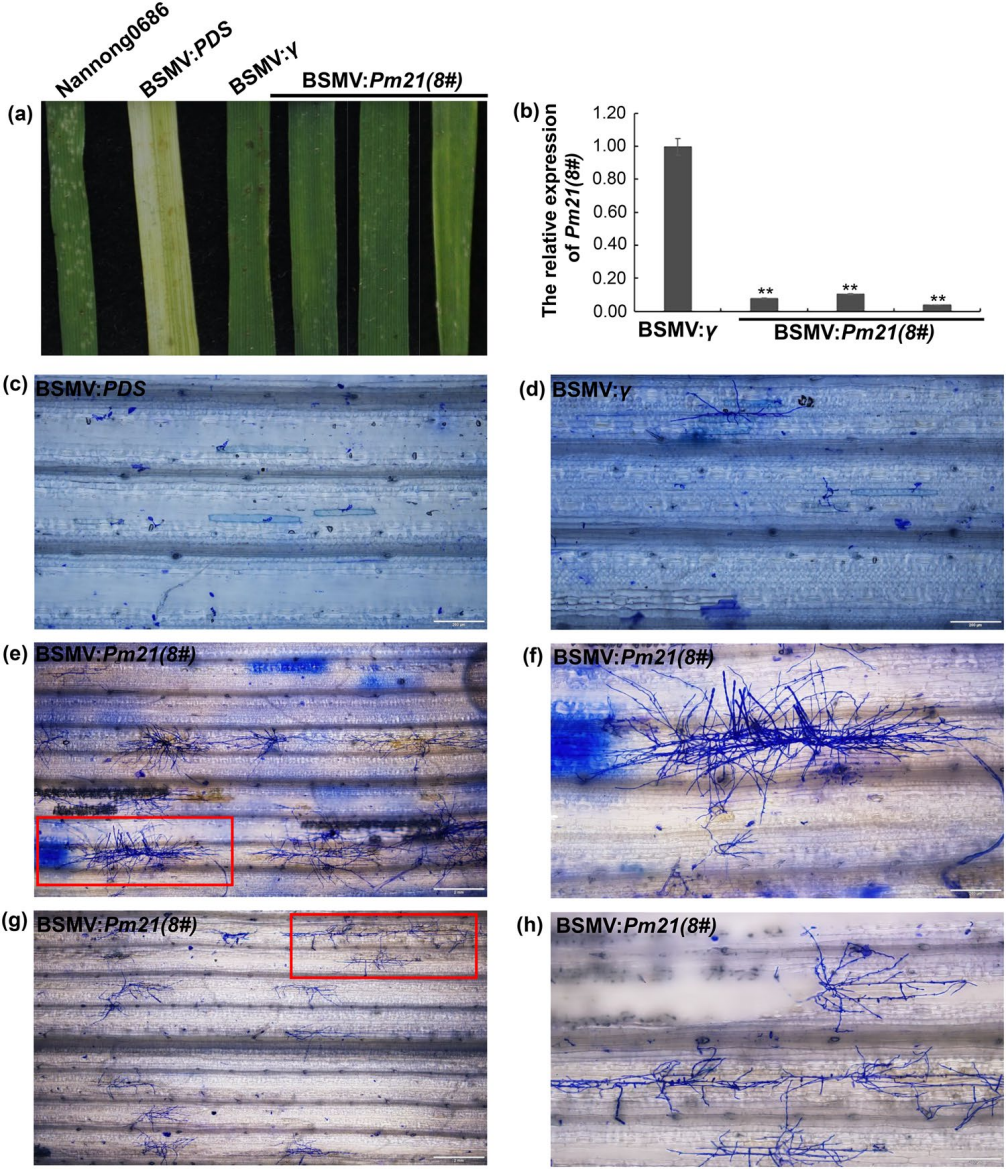
Functional analysis of *Pm21(8#)* using BSMV-VIGS, **a** Evaluation of *Bgt* resistance conferred by *Pm21(8#)* in T6VS(8#)·6DL by silencing of *Pm21(8#)*. Seven days after *Bgt* inoculation, no *Bgt* colonies were visible on the BSMV:*γ* infected leaves and on the BSMV:*PDS* infected leaves, while a small number of *Bgt* colonies were visible on the BSMV:*Pm21(8#)*-infected leaves, and a large number of *Bgt* colonies were visible in the susceptible control Nannong0686. **b** qRT‒PCR analysis of *Pm21(8#)* expression levels in both BSMV:*γ* and BSMV: *Pm21(8#)*-infected individuals. The expression level of *Pm21(8#)* was decreased significantly in the BSMV:*Pm21(8#)* infected leaves. **c–h** Observation of *Bgt* development under the microscope. *Bgt* developed more slowly and produced fewer hyphal branches in the BSMV:*γ* and BSMV:*PDS* infected leaves than in the BSMV:*Pm21(8#)*-infected leaves (**c** and **d**). Moreover, a large number of conidial chains could be produced in the BSMV:*Pm21(8#)*-infected leaves but not in the BSMV:*γ* and BSMV:*PDS*-infected leaves (**e** and **g**). Figures **f** and **h** show enlarged views of the red rectangles in **e** and **g**, respectively. The white line represents the scale bar 200 μm

To further verify that *Pm21(8#)* is a functional allele of *Pm21*, a probe that was previously used to silence *Pm21(4#)* in T6VS(4#)·6AL was used to silence *Pm21(8#)* in T6VS(8#)·6DL by virus gene induced gene silencing (VIGS) mediated by BSMV. The expression level of *Pm21(8#)* in T6VS(8#)·6DL was significantly decreased based on qRT‒PCR analysis (Fig. 3b). The results indicated that silencing of *Pm21(8#)* could completely abolish the resistance of T6VS(8#)·6DL, and a small number of colonies could be observed on the leaf surface (Fig. 3a). Microscopic observation also showed that *Bgt* produced many branches of hyphae and even conidiophores (Fig. 3c–h). This indicates that the resistance of T6VS(8#)·6DL is mediated by *Pm21(8#)* and that *Pm21(8#)* is a functional *Pm21* allele.

### Sequence analysis of *Pm21*(*8#*) with its haplotypes

#### Nucleotide sequence and gene structure of Pm21(8#)

We cloned the alleles of *Pm21* from 110 accessions of *H. villosa* using the primers designed according to the *Pm21(4#)* sequence that we previously reported (Xing et al. 2018), and 99 alleles corresponding to the coding region and 76 alleles corresponding to the promoter were successfully cloned. Sequence analysis indicated that there were high polymorphisms in both the gene coding region and in the promoter region. Using primers specific to *Pm21(*4#*)*, NLR1-F (5′-ATTGAGATGTCTGCACCGGTCG-3′) and NLR1-R (5′-CTCTCTTCGTTACATAATGTAGTG-3′), the genomic sequences of *Pm21(5#)* from T6VS(5#)·6DL were cloned, but the amplification of *Pm21(8#)* from T6VS(8#)·6DL failed, implying the existence of polymorphism between *Pm21(8#)* and *Pm21(*4#*)*. Then, another pair of primers, NLR1-F1 (5′-CTACAACCGCATCCTCAATCATACT-3′) and NLR1-R1 (5′-TTAAAGTAAAACTGGGACCACATT-3′), were designed based on the 5′-UTR and 3′-UTR region of *Pm21(*4#*)*, and the genomic sequence of *Pm21(8#)* was amplified and cloned. Sequence diversity was widespread in the coding region of genes (Fig. S3). At the same time, significant differences were also identified in the promoter sequences and *cis*-elements arrangements (Fig. S4a, S4b). The genomic sequence comparison showed that *Pm21(8#)* displayed 89.2% homology with *Pm21(4#)* and 90.0% homology with *Pm21(5#).*

**Fig. 4:**
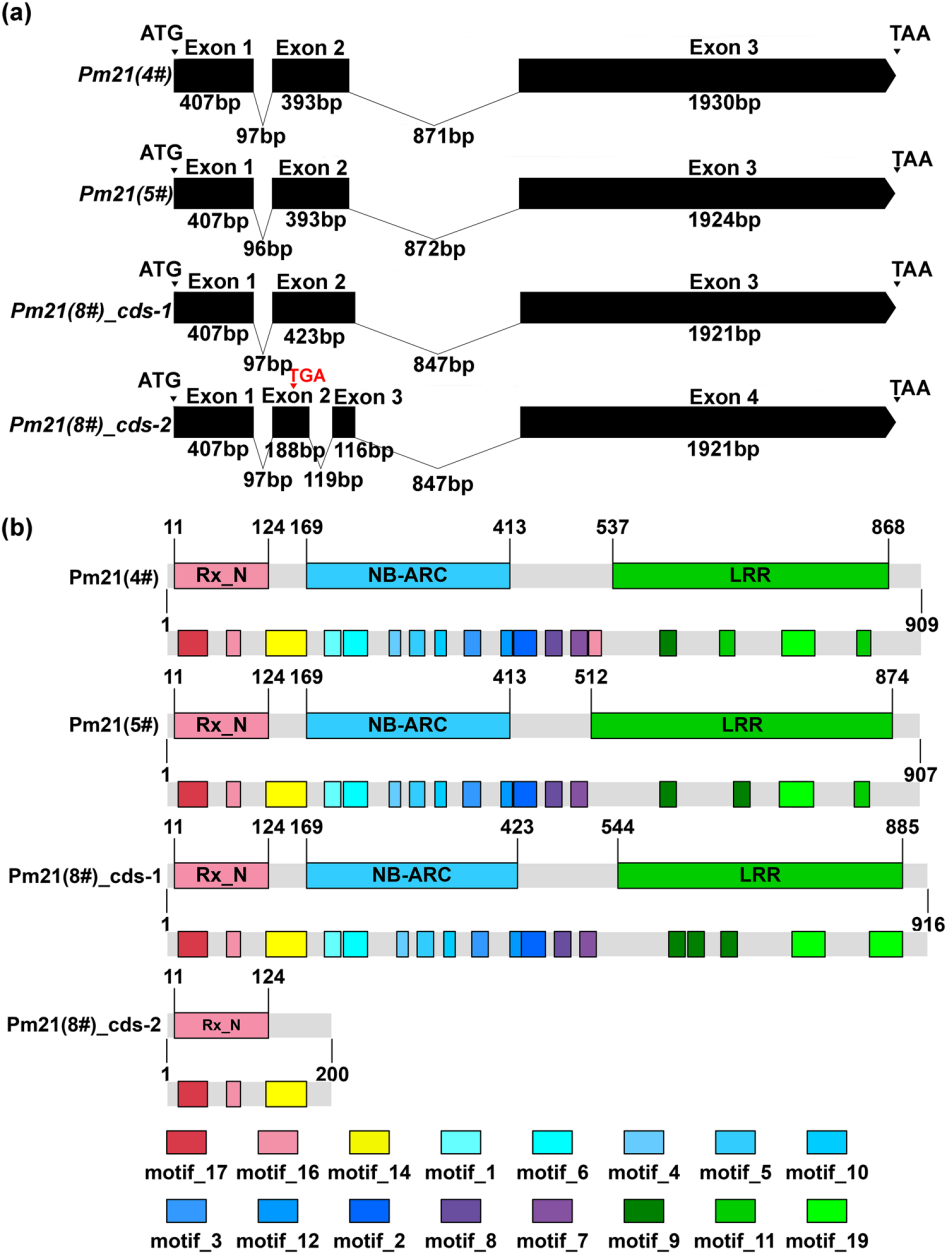
Comparison of the gene structures and protein compositions of the *Pm21(4#), Pm21(5#)* and *Pm21(8#)*, **a** Gene structures of *Pm21(4#)*, *Pm21(5#)* and *Pm21(8#)* by alignment of the genomic sequences with the corresponding transcripts. An alternative splicing pattern was identified in *Pm21(8#)* but not in *Pm21(4#)* and *Pm21(5#)*. Exons are represented by black rectangles, and introns are represented by lines. Their sizes are marked in bp. **b** Protein compositions of Pm21(4#), Pm21(5#), and Pm21(8#) based on searching the conserved motifs. Pm21(4#), Pm21(5#) and Pm21(8#)-1 were complete NLR proteins, while Pm21(8#)-2 was a truncated protein with a premature stop codon at 200 aa. The Rx_N (CC), NB-ARC and LRR domains were identified respectively, and each conserved motif distributed in the three domains is displayed in a different color (color figure online)

The CDS sequence of *Pm21(5#)* was amplified from T6VS(5#)·6DL using NLR1-F (5′-ATTGAGATGTCTGCACCGGTCG-3′) and NLR1-R (5′-CTCTCTTCGTTACATAATGTAGTG-3′), and *Pm21(8#)* was amplified from T6VS(8#)·6DL using NLR1-F1 (5′-CTACAACCGCATCCTCAATCATACT-3′) and NLR1-R1 (5′-TTAAAGTAAAACTGGGACCACATT-3′). Only one type of transcript corresponding to *Pm21(5#)* was identified, while two types of transcripts corresponding to *Pm21(8#)* were identified. In our previous study, we identified only one type of transcript corresponding to *Pm21(4#)* (Xing et al. 2018). The alignment of the genomic sequence with the transcripts indicated that the splicing pattern of *Pm21(8#)* was different from those of *Pm21(4#)* and *Pm21(5#)*. *Pm21(4#)* and *Pm21(5#)* showed only one type of splicing pattern and contained three exons and two introns, while *Pm21(8#)* presented two types of splicing patterns. The splicing pattern of *Pm21(8#)-1* was the same as those of *Pm21(4#)* and *Pm21(5#)*, while *Pm21(8#)-2* differed in the presence of one more intron in exon 2 (Fig. 4a, Fig. S6a). The RT‒PCR primers were designed such that one primer was located in exon 2 and the other located in exon 3 using samples of T6VS(8#)·6DL inoculated with *Bgt* for different times, and the results showed that two bands corresponding to *Pm(8#)-1* and *Pm(8#)-2* were produced, indicating that the alternative splicing did in fact occur in *Pm(8#)* (Fig. S6b). Then, qRT‒PCR was performed to analyze the expression pattern of *Pm(8#)-1* and *Pm(8#)-2*, and it was found that both transcripts could be induced by *Bgt* infection (Fig. S6c).

### Amino acid sequence comparison of Pm21(8#) with Pm21(4#) and Pm21(5#)

The putative amino acid sequences of Pm21(4#), Pm21(5#) and Pm21(8#) differ in length; Pm21(4#), Pm21(5#), and Pm21(8#)-1 contain 909, 907, and 916 aa, respectively, while Pm21(8#)-2 contains 200 aa due to frameshift causing premature termination by introducing a stop codon (Fig. 4b). Pm21(4#), Pm21(5#) and Pm21(8#)-1 were also scanned to identify the conserved motifs found in NLR proteins using Pfamscan and Interproscan. In the N-terminus, three proteins shared a high level of conservation. The regions corresponding to the amino acids 1–413 of Pm21(4#), 1–413 of Pm21(5#) and 1–423 of Pm21(8#) contained a coiled-coil and NB-ARC domains, with identical motif compositions and arrangements between these proteins. An extra motif_16 was present in the linker region of Pm21(4#) but not in the regions of Pm21(5#) and Pm21(8#). In the C-terminus, the conservation of the motif composition was relatively low; Pm21(4#) consists of Motif_9, Motif_19 and two Motif_11, Pm21(5#) consists of Motif_11, Motif_19 and two Motif_9, while Pm21(8#)-1 consists of three Motif_9 and two Motif_19 (Fig. 4b). Furthermore, the sequence identity of Pm21(8#) with Pm21(4#) is 90.1% and that with Pm21(5#) is 92.7% in the CC-NB-ARC region, while the sequence identity of Pm21(8#) with Pm21(4#) is only 79.0%, and that with Pm21(5#) is 81.7% in the LRR region.

### Motif comparisons among all *Pm21* alleles

Using the primers specific to *Pm21(4#)*, 101 sequences from 100 *H. villosa* accessions were cloned. The CDSs of all the alleles were predicted based on the splicing pattern of *Pm21(4#)*, and the corresponding protein sequences were obtained. In total, 70 full-length proteins were predicted with both NBS-ARC and LRR domains, 30 intact proteins were predicted to show only NBS-ARC but no LRRs, and one abnormal protein was predicted to show only 13 amino acids. Then, the redundant proteins were removed and 33 unique proteins were used to construct the phylogenetic tree (Fig. S7). From the diagram of protein compositions, it was found that the NB-ARC domains were highly conserved, while the LRR domains were highly diverse. The tree showed that all *Pm21* alleles were divided into three branches, among which Pm21(4#) belonged to the type I, while Pm21(5#) and Pm21(8#) belonged to type III (Fig. S7). It was also found that the protein compositions of Pm21(8#) were unique among all the alleles.

### Microscopic observation of the pathogen development in different *Pm21* haplotype backgrounds

**Fig. 5:**
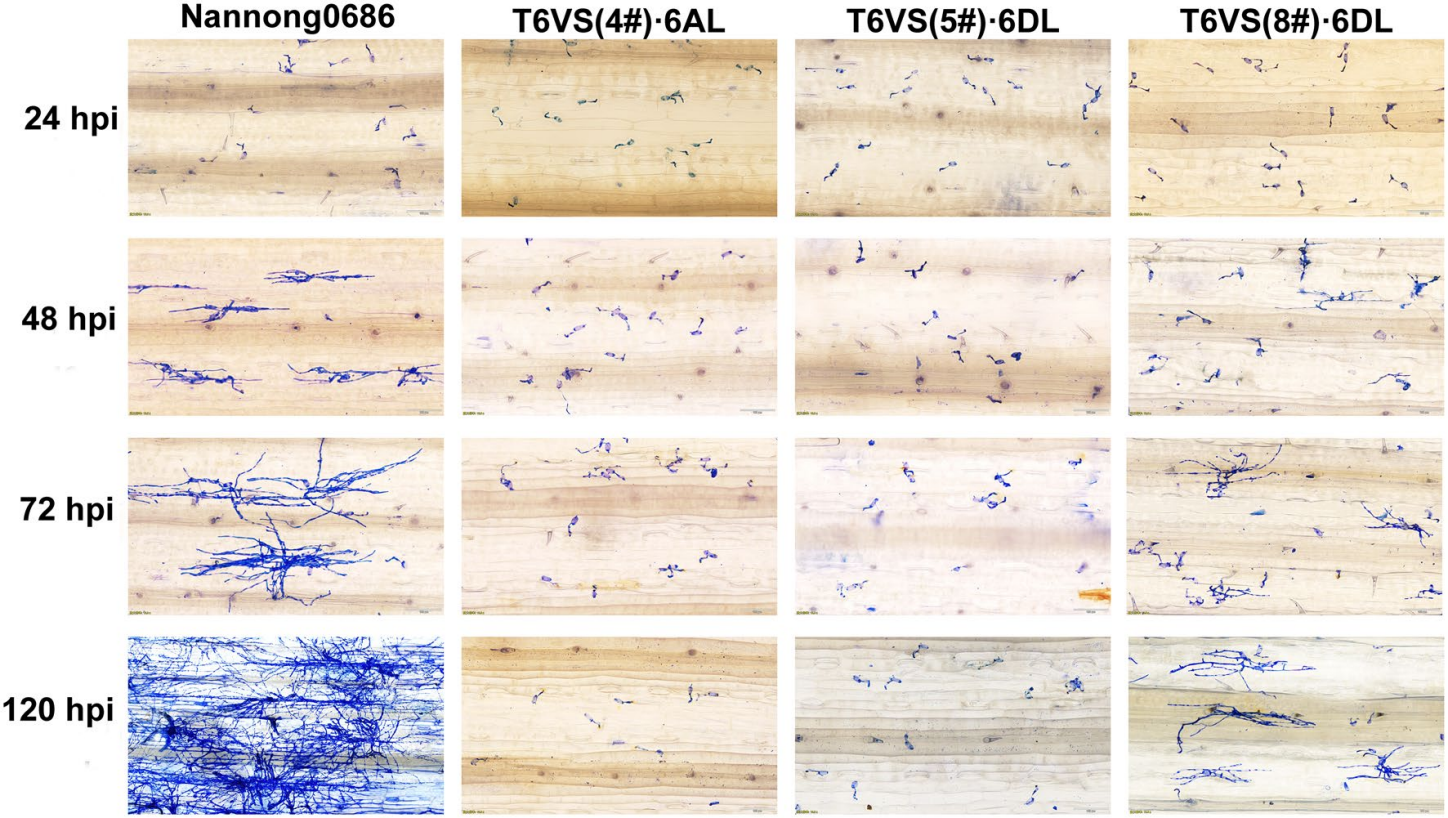
Observation of the *Bgt* development on the leaves of the three translocation lines at different time points after *Bgt* inoculation, An obvious difference was detected in T6VS(8#)·6DL, in which more conidia could produce hyphae. On the left side of the figure, 24 hpi, 48 hpi, 72 hpi and 120 hpi are the hours post *Bgt* inoculation, respectively. The white line represents the scale bar 100 μm

Although T6VS(8#)·6DL was as resistant to *Bgt* as T6VS(4#)·6AL and T6VS(5#)·6DL, it was interesting to study whether there was a difference in the resistance process in T6VS(8#)·6DL because of the distinct transcriptional and motif diversity of *Pm21(8#)*. The *Bgt* development on the leaves of the three lines was observed under a microscope, and an obvious difference in hyphal growth was detected in T6VS(8#)·6DL in the initial *Bgt* infection stage (Fig. 5). Then, the percentage of conidia producing secondary hyphae on the leaves of the three lines were statistical analyzed at 48 hpi. *Pm21(4#)* in T6VS(4#)·6AL and *Pm21(5#)* in T6VS(5#)·6DL showed stronger ability to inhibit the formation of the haustoria and the development of the hyphae than that of *Pm21(8#)* in T6VS (8#)·6DL (Fig. S9). Although *Pm21(8#)* showed weaker ability to inhibit the formation of haustoria and the development of hyphae, it reduced the growth of hyphae and inhibited the formation of conidiophores (Fig. 5) based on the comparison of *Bgt* development in T6VS(8#)·6DL and Nannong0686. This indicated that the resistance mechanism mediated by *Pm21(8#)* was different from that of *Pm21(4#)* and *Pm21(5#).*

Because genetic backgrounds of T6VS(5#)·6DL and T6VS(8#)·6DL are different, it needs to study whether the difference between *Pm21(5#)* and *Pm21(8#)* was induced by an element of the genetic backgrounds other than the *Pm21* haplotypes themselves. The polymorphic molecular markers, Pm21_588, Pm21_1178, and Pm21_2683, were developed (Fig. S5) for identifying the *Pm21(5#)* homozygous lines and *Pm21(8#)* homozygous lines from the F_2_ population crossing between T6VS(5#)·6DL and T6VS (8#)·6DL (Fig. S8). Then, the randomly selected lines from the *Pm21(5#)* homozygous lines, and *Pm21(8#)* homozygous lines were used to compare the *Bgt* development process. The development of *Bgt* in the *Pm21(5#)* homozygous lines was similar as that in T6VS(5#)·6DL, and the development of *Bgt* in the *Pm21(8#)* homozygous lines was similar to that in T6VS(8#)·6DL (Fig. S10). Therefore, it was verified that *Pm21(8#)* inhibited pathogen development in a distinct pattern, different from that of *Pm21(4#)* and *Pm21(5#)*.

### Analysis of the haustorium index in a single-cell *GUS*-expression assay

To further examine whether the ability to inhibit haustorium formation by *Pm21* haplotypes was different, single-cell transient expression of *GUS* gene was performed to statistically analyze the percentage of haustorium formation in the *Pm21(5#)* and the *Pm21(8#)* genetic backgrounds based on counting the visible haustoria in the *GUS*-expressed cells. The haustorium index in the susceptible Nannong0686 line was 53.6%, the haustorium indexes in Nannong9918 with *Pm21(4#)*, and in Nannong0686-*Pm21(5#)* and F_2_-*Pm21(5#)* were similar, while the haustorium indexes in Nannong0686-*Pm21(8#)* and F_2_-*Pm21(8#)* were significantly higher than those of materials containing *Pm21(4#)* and *Pm21(5#)* (Fig S11). Therefore, the observation of *Bgt* development and the statistical analysis of the haustorium index indicated that the ability of *Pm21(8#)* to prevent haustorium formation was different from that of *Pm21(4#)* and *Pm21(5#)*.

### ROS-inducing activity of different *Pm21* haplotypes

**Fig. 6:**
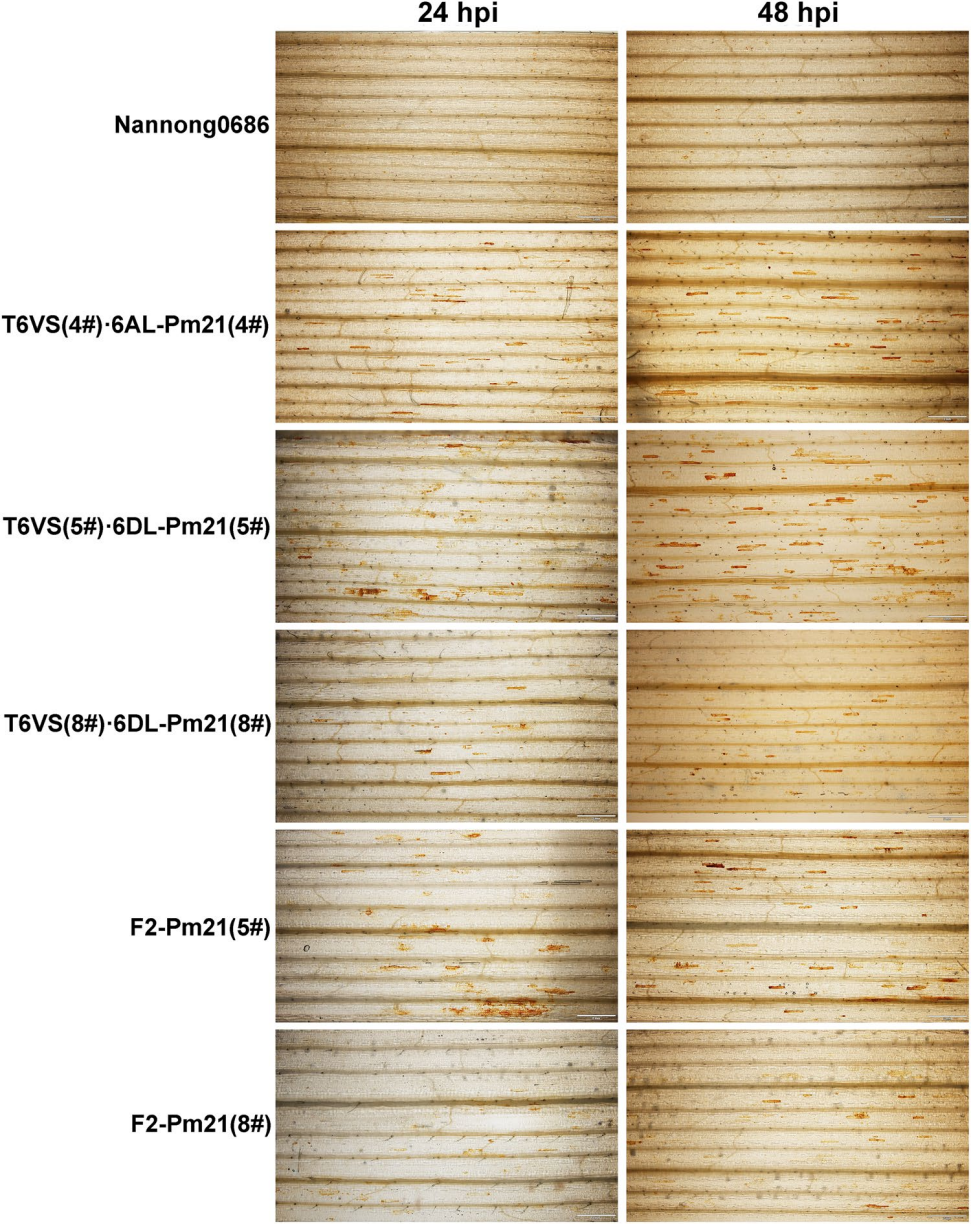
Detection of ROS accumulation by DAB staining of *Bgt*-infected leaves, An obvious difference was detected in materials containing *Pm21(8#)*, including T6VS(8#)·6DL and F_2_-Pm21(8#), in which fewer *Bgt*-interacting cells showed ROS accumulation. In the up portion of the figure, 24 hpi and 48 hpi are the hours post *Bgt* inoculation. The white line represents the scale bar 2 mm

To test the ROS-inducing activity of different *Pm21* haplotypes, ROS accumulation at the *Bgt* interaction sites was observed at 24 hpi and 48 hpi. It was found that ROS accumulation cells after *Bgt* inoculation were less in materials containing *Pm21(8#)* than in materials containing *Pm21(4#)* and *Pm21(5#)* (Fig. 6). Then, the ROS-induced activity was statistically analyzed in *Pm21(5#)*- and *Pm21(8#)-*containing materials, Nannong0686-*Pm21(5#)* *(T6VS(5#).6DL-Pm21(5#))* and Nannong0686-*Pm21(8#) (T6VS(8#).6DL-Pm21(8#))*, at 24 hpi and 48 hpi. Two types of *Bgt-*interacting cells were observed; one type with ROS accumulation around the penetration pegs (Type a) and the other type with ROS accumulation in the whole *Bgt*-interacting cell (Type b) (Fig. S12 a, b). The numbers of *Bgt* corresponding to ‘Type a’ and ‘Type b’ cells were counted, and the ratio of ROS-producing *Bgt* spores to the total number of germinated *Bgt* spores was statistically compared. The ROS-inducing activity of Nannong0686-*Pm21(8#)* and F_2_-*Pm21(8#)* was significantly lower than that of Nannong0686-*Pm21(5#)* and F_2_-*Pm21(5#)*. (Fig. S12 c, d). Therefore, it was indicated that the ROS-inducing activity observed after the *Bgt* infection of *Pm21(8#)* was lower than that observed after the *Bgt* infection of *Pm21(5#)*, and this difference was not produced by the genetic background.

## Discussion

### Different *Pm21* haplotypes regulate powdery mildew resistance by inhibiting *Bgt* growth at different developmental stages

The *Bgt* infection process in leaves includes primary tube germination, attached tube germination, appressorium formation, penetration peg development in the tip of appressorium, penetration peg entry into epidermal cells, haustorium formation under the penetration peg, primary hyphae development, secondary penetration peg and secondary haustorium formation under the primary hyphae, secondary hyphae development into large colonies with many branches of hyphae (Glawe 2008). If a conidial chain forms and new conidia are released, there will be a second (or more) round of infection. In the leaves of susceptible Nannong0686, a large number of haustoria formed in the early infection stage, and white colonies produced due to the high density of hyphae and conidia chains in the later infection stage. *Pm21(4#)* and *Pm21(5#)* inhibited the formation of haustoria strongly, leading to very few hyphal production. Most powdery mildew resistance genes play a role in disease resistance by inhibiting the formation of haustoria (Zou et al. 2018; Hurni et al. 2013; Xie et al. 2020) However, the haustorium inhibition activity of *Pm21(8#)* was significantly lower than those of *Pm21(4#)* and *Pm21(5#)*. So, how does *Pm21(8#)* exert disease resistance? Hyphal development was inhibited by *Pm21(8#)* leading to a reduced number of branches, and no conidial chain was formed; thus, no visible white colonies could be observed, and no further rounds of infection could be induced. For inhibiting *Bgt* development, *Pm21(8#)* works mainly at the late secondary hyphae producing stage, but *Pm21(4#)* and *Pm21(5#)* works mainly at the early haustoria formation stage. Therefore, it was suggested that the resistance mechanism of *Pm21(8#)* was different from that of *Pm21(4#)* and *Pm21(5#).*

### The LRR domain of Pm21 haplotypes affects the resistance patterns

Because of the rapid variation in pathogens, maintaining the diversity of NLRs is necessary for plants to withstand the evolutionary battle (Baggs et al. 2017). A number of studies have revealed that natural variations in protein structures and copy number of the NLR genes could change the resistance spectrum to plant pathogens, especially when the mutations and expansions occur in LRR domains. The LRR domains in NLR genes have long been speculated to be involved in determining the plant’s ability to recognize specific pathogen effectors (DeYoung et al. 2006). In maize (*Zea mays* L.), variations in the LRR domains of the *Rp1* locus could alter race-specific resistance to rust (Collins et al. 1999; Sun et al. 2001). Similarly, variations in the LRR domains of the *Pm3* and *Pm60* loci in wheat and the *Mla* locus in barley (*Hordeum vulgare* L.) were tightly related to the resistance spectrum to powdery mildew (Bhullar et al. 2009; Seeholzer et al. 2010; Zou et al. 2022). In the last 20 years, the structure of the R protein has been analyzed to identify important protein domains and key amino acid sites, which has facilitated the molecular design of new variations to obtain new resistance genes. It has been reported that the artificial mutation of NBS or the LRR region of NLR resistance proteins, such as RX and R3a, could change the recognition of effectors and expand the resistance spectrum (Harris et al. 2013; Chapman et al. 2014; Segretin et al. 2014; Stirnweis et al. 2014). Natural diversity of the LRR domains in different *Pm21* haplotypes was found in this study, which was conjectured to affect the resistance pattern. Therefore, the resistance mechanism of the *Pm21* haplotypes should be identified.

### ROS accumulation influenced by *Pm21* may be tightly related to the resistance signal

In recent years, with the rapid development of high-throughput sequencing technology, molecular biology and bioinformatics, an increasing number of *Pm* genes have been cloned. Among the successfully cloned *Pm* genes, most of them encode NLR-type proteins, except that *Pm38* encodes an abscisic acid transporter, *Pm46* encodes a hexose transporter, *Pm24* encodes a tandem kinase and *Pm4a* encodes an MCTP-kinase (Krattinger et al. 2009; Moore et al. 2015; Lu et al. 2020; Sánchez-Martín et al. 2021)*.* The diversity of PM resistance proteins indicates that the struggle between wheat and powdery mildew pathogens involves a complex mechanism. ROS production not only is a phenomenon accompanying the interaction between plants and pathogens but also plays an important role in the plant defense response. ROS accumulation at infection sites and in whole cells was shown to be significantly different in *Pm21* haplotypes after *Bgt* inoculation, which indicates that the resistance effects and the underlying resistance mechanisms of *Pm21* haplotypes should be different. The powdery mildew fungus is an obligate biotrophic pathogen that extracts nutrients from plant host cells via its haustoria; therefore, the formation of haustoria is a crucial step of *Bgt* development in the host. In this study, the inhibition of haustoria formation by *Pm21(5#)* was stronger than by the inhibition mediated by *Pm21(8#)*, while the percentage of ROS accumulation was higher in *Pm21(5#)* than in *Pm21(8#)*. Therefore, we inferred that ROS accumulation induced by different *Pm21* haplotypes after *Bgt* infection might correlate with its ability to inhibit haustoria formation. However, the inhibition of hyphal growth and the suppression of further conidial chains formation were observed during the late *Bgt* infection period in *Pm21(#8)*-containing materials. It was speculated that different intercellular resistance regulation pathways were involved in *Pm21(#8)* in the post-haustorium formation stage, which needs to be investigated.

### Genetically diverse *Pm21* haplotypes and 6VS chromosome arms provide new resources for wheat resistance breeding

The large-scale and long-term use of a single or a few disease resistance genes makes it easy to lose resistance. Therefore, the exploration and utilization of genetically diverse disease resistance genes to control crop disease has attracted much attention for a long time. Multiple strategies have been adopted in wheat breeding to increase the durability of resistance genes, such as pyramiding diverse resistance genes in one variety, developing multiline varieties with different resistance genes in the same genetic backgrounds, the rational distribution and rotation of the valuable resistance genes, and the utilization of varieties with different resistance patterns (Brunner et al. 2012; Koller et al. 2018; Hafeez et al. 2021). These strategies can make full use of the diversity of disease resistance genes and have been used in disease resistance breeding. The wild relatives of wheat contain precious gene resources for increasing genetic diversity and belong to the tertiary gene pool for wheat genetic improvement (Qi et al. 2007; Li et al. 2016). The broad-spectrum resistance mediated by *Pm21(4#)* gene from *H. villosa* has powerful resistance effect and is easily selected in the field; therefore, overreliance on *Pm21(4#)* by breeders is responsible for an increasing risk of the resistance breakdown. In addition, all the varieties harboring *Pm21(4#)* contained the same chromosome arm 6VS(4#), thus reducing the genetic diversity of this chromosome. One of the most effective and economical approaches to combat the high genetic variability of pathogens is to efficiently mine and appropriately deploy broad-spectrum resistance gene haplotypes. In this study, a new chromosome arm 6VS(8#) with a novel *Pm21(8#)* haplotype was developed and transferred to wheat, which provides both diverse broad-spectrum resistance gene resource *Pm21(8#)* and genetic germplasm resources including 6VS(8#) for the improvement of disease resistance in the future.

## Supplementary Information

Below is the link to the electronic supplementary material.

Supplementary file1 (DOCX 106961 kb)

## Data Availability

The nucleotide sequences of complete CDS with two transcript splicing patterns and the genomic sequence for *Pm21(8#)* were submitted to NCBI GenBank with accession number: ON505440, ON505441, ON505442. The plant materials reported in the manuscript are freely available to all the readers on reasonable request.
