# Supplementary material for: Identification and transfer of a new Pm21 haplotype with high genetic diversity and a special molecular resistance mechanism
Source: Theor Appl Genet. 2023 Jan 19;136(1):10. doi: 10.1007/s00122-023-04251-y (PMC9852157; doi:10.1007/s00122-023-04251-y)
Supplement: Supplementary file 1 — Supplementary file1 (DOCX 106961 kb) [file 122_2023_4251_MOESM1_ESM.docx]

**Supplementary Information**

**Fig. S1 Cytogenetic comparison of the three wheat-*H. villosa* translocation chromosomes involved 6VS through oligo-painting**

(a, b, c) FISH analysis of T6VS(4#)·6AL, T6VS(5#)·6DL and T6VS(8#)·6DL using mixed oligonucleotide probes on root tip metaphase chromosomes. The green signals were produced by oligo-pSc119.2-1 and oligo-(GAA)10, and the red signals were produced by oligo-pAs1-1, oligo-pAs1-3, oligo-pAs1-4, oligo-pAs1-6, oligo-AFA-3 and oligo-AFA-4.

(d, e) The cropped translocation chromosomes, 6VS(4#)·6AL, T6VS(5#)·6DL and T6VS(8#)·6DL, from the GISH analysis using total genomic DNA of *H. villosa* as a probe (d) and cropped from the subsequent FISH analysis using mixed oligonucleotide probes (e). The 6VS chromosome arms showed similar green fluorescence in GISH analysis (d), while showed high polymorphism in FISH analysis (e).

**Fig. S2 Allelic testing of *Pm21(8#)* with *Pm21(5#)***

Evaluation of the powdery mildew resistance at the seedling stage with detached leaves of an F_2_ population derived by crossing between T6VS(5#)·6DL and T6VS (8#)·6DL. Wheat cv. Nannong9918 carrying *Pm21(4#)* was using as the resistant control, and wheat cv. Nannong0686 was used as the susceptible control.

**Fig. S3 Alignment of the genomic sequences of *Pm21(4#)*, *Pm21(5#)* and *Pm21(8#)***

**Fig. S4 Comparison of the promoter sequences of the three *Pm21* haplotypes**

Sequence alignment (a) and predicted *cis*-elements arrangements (a) in the promoter region of the three *Pm21* haplotypes.

**Fig. S5 Development of molecular markers specific to individual *Pm21* haplotypes**

The polymorphic molecular markers Pm21_588 was co-dominant and could distinguish *Pm21(4#)/Pm21(5#)* from *Pm21(8#)*, Pm21_1178 was dominant and could distinguish *Pm21(4#)* from *Pm21(5#)/Pm21(8#)*, Pm21_2683 was dominant and could distinguish *Pm21(5#)* from *Pm21(4#)/Pm21(8#)*. The arrows indicated the polymorphism bands.

**Fig. S6 Detection of the alternative splicing patterns of *Pm21(8#)***

(a) The alternative splicing occurring in the Exon 2 producing *Pm21(8#)-1* and *Pm21(8#)-2*

(b) RT‒PCR analysis to show the alternative splicers of *Pm21(8#)-1* and *Pm21(8#)-2* in the leaves of T6VS(8#)·6DL inoculated with *Bgt*. The primers were located to the Exon2 and Exon 3 of *Pm21(8#)-2*, respectively.

(c) qRT‒PCR analysis to show the expression level of *Pm21(8#)-1* and *Pm21(8#)-2* in the leaves of T6VS(8#)·6DL inoculated with *Bgt* using *TaTubulin* as the internal control.

**Fig. S7 Phylogenetic tree of 33 Pm21 haplotypes**

The phylogenetic tree was constructed using 33 non-redundant Pm21 proteins including Pm21(8#), Pm21(4#) and Pm21(5#) from more than 100 *H. villosa* accessions. The conserved motifs from each NLR were displayed using different colors in the protein structures.

**Fig. S8 Genotyping** **of the individual plants from the F_2_ population**

The polymorphic molecular markers, Pm21_588, Pm21_1178, and Pm21_2683, were used to identify the *Pm21(5#)* homozygous lines and *Pm21(8#)* homozygous lines from the F_2_ population derived from crossing between T6VS(5#)·6DL and T6VS (8#)·6DL.

**Fig. S9 Statistical analysis of the ability to inhibit conidia development**

(a)	Statistical analysis of the percentage of conidia producing secondary hyphae at 48 hpi.

(b) Statistical analysis of the number of conidia producing secondary hyphae per cm^2^ at 48 hpi. Different letters indicate significantly differences at P<0.05.

**Fig. S10 Observation of the *Bgt* development on the leaves of the *Pm21(5#) and Pm21(8#)* homozygous lines**

An obvious difference was detected in *Pm21(8#)* homozygous lines in that more conidia could produce hyphae. 24h, 48h, 72h and 120h represented hours after *Bgt* inoculation, repressively.

**Fig. S11 Analysis of the haustorium index based on** **single-cell GUS-expression assay**

(a)	Transient expression of GUS gene by bombardment for reporting the transformed cells and co-staining haustoria in the *Bgt* infected cell. *Bgt* development process was observed, geec cells with haustoria and without haustoria were shown in the figures. GUS expression epidermal cell (geec), conidium (con), penetration peg (pp), haustoria (ha), and secondary hyphae (sh).

(b) Statistical analysis of haustorium index in susceptible Nannong 0686, Nannong9918 carrying *Pm21(4#)*, Nannong0686-Pm21(5#) and Nannong0686-Pm21(8#) lines, F_2_-Pm21(5#) and F_2_-Pm21(8#) lines. Different letters indicate significantly differences at P<0.05.

**Fig. S12 Statistical analysis of ROS accumulation in the *Bgt*-interacting cells**

(a, b) *Bgt* induced ROS accumulation in the whole interacting cells (pointed with red arrows) or around the penetration pegs (pointed with black arrows). Figure a showed both types of interacted cells and Figure b showed the interacted cells full of ROS.

(c, d) The ratio of ROS-inducing *Bgt* to total germinated *Bgt* were statistically analyzed in Nannong0686-*Pm21(5#)* and Nannong0686-*Pm21(8#)* lines, F_2_-*Pm21(5#)* and F_2_-*Pm21(8#)* lines at 24 hpi and 48 hpi. The percentage of ROS-inducing *Bgt* with ROS accumulation both in the whole interacting cells and around the penetration pegs was shown in figure c, while the percentage of ROS-inducing *Bgt* with ROS accumulation in the whole interacting cells was shown in figure d. The white line represents scale bar=200μm in Figure a, and the white line represents scale bar=100 μm in Figure b. Different letters in figure c and d indicate significantly differences at P<0.05.

| **TableS1. The sequences of primers used in this study.** | |
| --- | --- |
| **primer name** | **sequence(5'→3')** |
| NLR1-F | ATTGAGATGTCTGCACCGGTCG |
| NLR1-R | CTCTCTTCGTTACATAATGTAGTG |
| NLR1-F1 | CTACAACCGCATCCTCAATCATACT |
| NLR1-R1 | TTAAAGTAAAACTGGGACCACATTC |
| Pm21_588-F | CGGGGCTTGTTGGTATTGAT |
| Pm21_588-R1 | TTGCAACTCGTGAGCGTGAG |
| Pm21_588-R2 | CAAGTGCACAAGTGTCAGTTCG |
| Pm21_1178-F | GCGCCACTAGCAGAAAGAAT |
| Pm21_1178-R1 | ATAAGTAATATCTGATAGATGGCCC |
| Pm21_1178-R2 | CCGAAGTCAAGAAAGTAGAAGTT |
| Pm21_2683-F | ACCTTCGGGTGCTAGTGTTT |
| Pm21_2683-R1 | GGTATGCTTTGTGTGAAACCAGA |
| Pm21_2683-R2 | ACCCGCCACCACTCTAGTAG |
| RT-PCR-R | TACTCTACTTCCATTCCCGAC |
| RT-PCR-cds1-F | AAGGCTCCTTGGTGGTGTC |
| RT-PCR-cds2-F | GGATTTGGAGGTCTAGGAGC |
| SQRT-PCR-nPm21-F1 | TGGCAATCTACAAGCAGGCA |
| SQRT-PCR-nPm21-R1 | TCTTGCAACTCGTGAGCGT |
